# Intrauterine device migration into the bladder with resultant bladder stone formation: a case report

**DOI:** 10.3389/fmed.2026.1931569

**Published:** 2026-09-03

**Authors:** Meilian Chen, Hongmei Xiao, Fei Lai, Yingxue Bai, Yixue Zhou, Xuelian Tan, Limei Wu, Haixia Jiang

**Affiliations:** 1 Department of Obstetrics and Gynecology, West China School of Medicine, Sichuan University Affiliated Chengdu Second People's Hospital, Chengdu Second People's Hospital, Sichuan University, Chengdu, China; 2 Department of Urology, West China School of Medicine, Sichuan University Affiliated Chengdu Second People's Hospital, Chengdu Second People's Hospital, Sichuan University, Chengdu, China

**Keywords:** bladder stones, intrauterine device, laparoscopic bladder repair, migration, transurethral bladder lithotripsy

## Abstract

**Objective:**

To investigate the possible aetiology, clinical characteristics, diagnostic and therapeutic approaches, and prognosis of intrauterine device (IUD) migration into the bladder.

**Case report:**

A 39-year-old woman presented with recurrent symptoms of urinary urgency and dysuria. Computed tomography scans (CT) revealed that one arm of the IUD had migrated into the bladder, causing bladder stones. The patient underwent transurethral bladder lithotripsy and stone removal, laparoscopic bladder repair, and hysteroscopic removal of the IUD. The IUD was completely removed, and the bladder stones were successfully treated. The patient made a good postoperative recovery.

**Conclusion:**

Regular follow-up examinations are required following the insertion of an IUD, with ultrasound being the preferred method of follow-up. If recurrent urinary symptoms occur, the possibility of IUD migration into the bladder should be ruled out. CT can assist in the diagnosis.

## Background

Intrauterine devices (IUDs) are widely used because of their low cost, safety, effectiveness, and reversibility, and they are the primary method of contraception for women in China who do not wish to conceive (1–3). IUD insertion is an invasive procedure, and surgical and postoperative complications are inevitable. Common complications include infection, pain, bleeding, and dysmenorrhoea, while the incidence of tissue adhesions and organ damage caused by IUD migration ranges from 0.1 to 0.9% (2, 4, 5). This study reports the clinical data of a patient admitted to our hospital in whom one arm of an IUD became embedded in the bladder, leading to the formation of bladder stones. We analyse the causes, diagnosis, and treatment, with the aim of providing a reference for the future clinical management of IUD migration into the bladder.

## Case report

A 39-year-old woman (gravida 1, para 1) wishing to conceive had her IUD removed at a local hospital 2 days prior to admission. A preoperative ultrasound scan revealed a hyperechoic IUD within the uterine cavity, with part of it protruding through the left anterior wall of the lower uterine segment beyond the serosa and into the bladder wall. Part of the bladder wall was obscured by the acoustic shadow of a stone located anteriorly, resulting in an unclear image. IUD impaction was suspected, and the possibility of IUD migration into the bladder could not be ruled out, the patient was therefore admitted to our hospital. The patient reported occasional urinary urgency and dysuria for more than 2 years, but no symptoms of urinary frequency, haematuria, abdominal pain, bloating, or irregular vaginal bleeding. She had a history of caesarean section 16 years previously, and an IUD had been inserted more than 10 months after the caesarean section (15 years ago). She reported no particular problems during or after the procedure.

**Figure 1:**
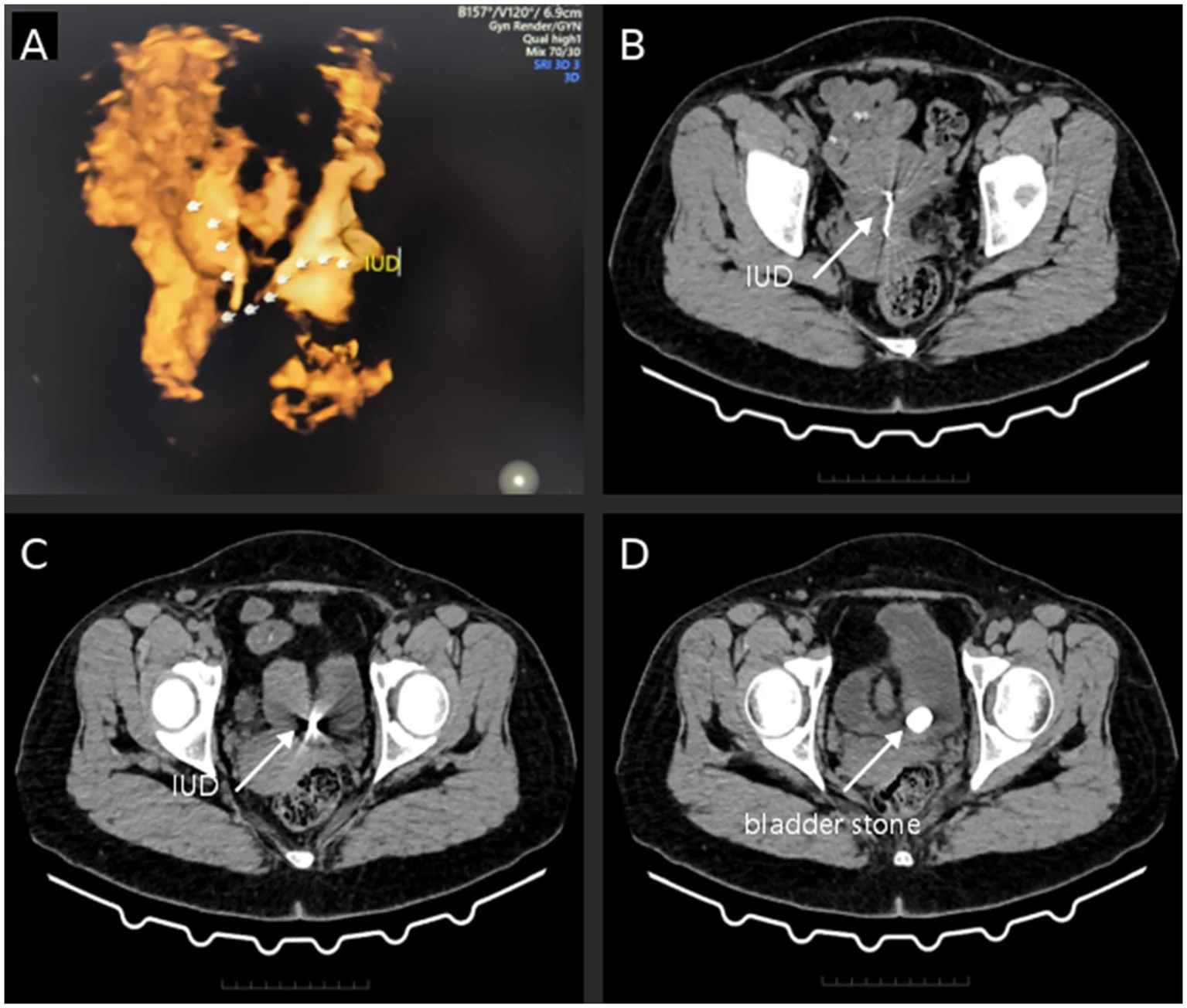
Preoperative ultrasound and CT findings demonstrating IUD migration into the bladder with secondary bladder stone formation. **(A)** Ultrasonography demonstrating the majority of the IUD within the endometrial cavity, with one arm penetrating the myometrium and projecting toward the bladder. **(B)** Axial CT image revealing a linear hyperdense IUD (white arrow) within the uterine cavity. **(C)** Axial CT image demonstrating one IUD arm (white arrow) traversing the uterine wall and entering the bladder lumen. **(D)** Axial CT image showing a hyperdense bladder calculus (white arrow) encasing the distal IUD arm.

Following admission, a gynaecological examination revealed no abnormal findings, and there was no bleeding or discharge from the vagina. Abdominal examination revealed no tenderness, muscle rigidity or rebound tenderness. The patient was admitted with a diagnosis of an impacted IUD; the possibility of device displacement cannot be ruled out. A repeat transvaginal ultrasound examination at our hospital revealed a hyperechoic IUD in the lower uterine segment in an abnormal position. One of its arms appeared to have penetrated the serosa through the anterior wall incision and reached the bladder wall. The relationship with the bladder wall was unclear, making it impossible to determine whether it had perforated the bladder wall. Several hyperechoic masses with acoustic shadows, the largest measuring approximately 1.8 cm, were observed within the bladder at this site (Figure 1A). Given inconclusive ultrasonographic findings, an embedded IUD complicated by bladder calculi could not be excluded. As the device was incompletely visualised, precise preoperative localisation was not attainable, consequently, computed tomography (CT) was undertaken to further delineate the position of the IUD. A subsequent pelvic CT scan revealed that one arm of the IUD had become displaced and penetrated the bladder (Figures 1B,C), with localised stone formation (Figure 1D). Routine urinalysis showed 165.9 white blood cells per microlitre, whilst urine culture identified *Escherichia coli* with a colony count of >10^5^ CFU/mL and positive extended-spectrum β-lactamase production. A consultation with the urology department was sought. Following 1 week of antimicrobial therapy based on susceptibility testing, repeat urinalysis showed 42.8 white blood cells per μL, whilst urine culture was negative for bacteria.

Based on the CT scan results, the surgical approach was decided in consultation with a urologist. Minimally invasive surgery is the preferred modality for managing IUD displacement, however, the specific approach must be tailored to the individual patient’s clinical circumstances and reproductive goals. As the patient was 39 years of age and desired future fertility, transabdominal incisions involving the bladder and uterus were considered excessively invasive and potentially detrimental to reproductive outcomes. Following preoperative discussions, it was decided to proceed with urological surgery first. Complete encasement of the IUD arm within the bladder stone precluded primary hysteroscopic extraction, as this approach carried an unacceptably high risk of device fragmentation, incomplete retrieval, and catastrophic injury to the bladder and surrounding pelvic structures. A staged multidisciplinary procedure was planned: (i) transurethral cystolitholapaxy for stone fragmentation and evacuation; (ii) laparoscopic retrieval of the migrated IUD segment with concomitant bladder repair; and (iii) hysteroscopic removal of the remaining device, followed by laparoscopic uterine wall repair at the perforation site.

On the 8th day after admission, transurethral bladder lithotripsy and stone removal, laparoscopic bladder repair, and hysteroscopic removal of the IUD were performed. Intraoperatively, multiple round, hard stones were observed on the posterior wall of the bladder, encircling the IUD. The largest stone measured approximately 1.5 cm in diameter and was surrounded by flocculent material and necrotic tissue. The bladder mucosa was congested. No masses were observed, the openings of both ureters were in their normal positions, and no active bleeding was noted. The stones were fragmented using a holmium laser at an energy setting of 2.0 J × 20 Hz to expose the IUD. Laparoscopic dissection was then performed to separate the adhesions between the uterus and the bladder. Following removal of the IUD from the bladder, bladder repair was carried out. Further hysteroscopic examination revealed a V-shaped IUD in the lower uterine segment, with one arm of the device protruding through the left anterior wall. The IUD was completely removed under direct hysteroscopic visualisation. Postoperative antimicrobial therapy was administered, and the patient recovered well and was discharged. A urinary catheter was left in place for 10 days. Upon returning to the hospital for catheter removal, the patient was able to void spontaneously without difficulty and reported no discomfort. At the 6-month and 12-month follow-up, she remained asymptomatic, with normal urinalysis and pelvic ultrasound findings.

## Discussion

The primary route of IUD migration is through the uterine wall into the pelvic and abdominal cavities. A rare route involves passage through the fallopian tubes into the pelvic and abdominal cavities. In this case, one arm of the V-shaped IUD had penetrated the serosal layer at the caesarean section scar and subsequently perforated the bladder wall. The probable cause was that the IUD was inserted while the patient was breastfeeding (6, 7). Although the patient had resumed menstruation more than 10 months postpartum, she was still breastfeeding, and the uterine myometrium may have remained thin and soft. If the surgeon did not correctly assess the position, size, and orientation of the uterus, this could have resulted in procedural error, increasing the risk of uterine perforation and IUD migration. Furthermore, the prolonged duration of IUD placement may also have contributed to its migration (8). In this case, the patient had had the IUD in place for 15 years. Because she experienced no discomfort immediately before or after insertion, it is presumed that the IUD was initially positioned correctly. However, over time, the IUD may have gradually become embedded in the myometrium, eventually penetrating the serosal layer at the caesarean section scar in the lower uterine segment, becoming embedded in and displaced into the bladder. Stones subsequently formed around the displaced IUD within the bladder, thereby causing urinary symptoms such as urgency and dysuria. Previous studies have found that caesarean section also increases the likelihood of IUD migration (9). Previous studies have also shown that the AiMu MCu series of IUDs are prone to migration (10). It is therefore hypothesised that the migration of the IUD in this patient resulted from multiple contributing factors. This study further confirms that caesarean section and the insertion of the AiMu MCu series IUD are high-risk factors for IUD displacement, while factors such as the patient being in the lactation period at the time of insertion, improper insertion technique, and the IUD remaining in the uterine cavity for an excessively long period are potential causes of IUD displacement.

Following IUD insertion, patients are advised to undergo regular imaging examinations, with ultrasound being the preferred modality. However, in cases of IUD migration, ultrasound is unable to visualise the entire shape of the device. Furthermore, because it is influenced by patient positioning and has limited resolution for non-copper-containing IUDs, misdiagnosis is common (11). In this case, ultrasound suggested an impacted IUD; however, it was not possible to determine the relationship between the device and the bladder, and CT was therefore performed.

CT provides clear imaging of high-density structures and allows reconstruction of sagittal and coronal images. It can clearly demonstrate the shape and position of the IUD, as well as the relationship between the displaced IUD and surrounding tissues and adjacent organs (4). Furthermore, because CT is not affected by patient position or intestinal peristalsis, it provides a more intuitive visualisation of a displaced IUD within the pelvic cavity. In this case, CT had clearly identified the position of the IUD. Therefore, the surgical approach was determined in consultation with the urology department before surgery to avoid complications associated with blind hysteroscopic removal, such as failure to remove the device, IUD fracture, post-removal vesicocervical fistula, and residual bladder stones.

For IUDs embedded in the uterine myometrium, hysteroscopic surgery is the treatment of choice. However, once migration of the IUD into the pelvic cavity is detected, early laparoscopic or open surgery is recommended, with laparoscopy being the preferred approach. Nevertheless, there is a 25% probability that laparoscopic surgery will require conversion to open surgery because of adhesions or other factors. Depending on the specific site of migration, multidisciplinary consultation is recommended, and the relevant departments should participate in the surgical management. In this case, following the discovery of IUD migration, the patient underwent thorough preoperative preparation and received anti-infective treatment. Through collaboration between our department and the Department of Urology, a combined hysteroscopic and laparoscopic procedure was performed, resulting in complete removal of the displaced IUD and successful treatment of the bladder stones using a holmium laser. The patient’s prognosis is favourable.

Although single-case reports have certain limitations that affect the generalisability of their conclusions, this case report nevertheless provides many important insights for clinical practise. In this study, we systematically discussed the possible causes of the IUD migration into the bladder in this patient and clearly demonstrated the relationship between the displaced IUD and the surrounding tissues and adjacent organs using ultrasound and CT scans. As the majority of the patient’s IUD was located within the uterine cavity, imaging examinations effectively helped to avoid serious complications that might have arisen during blind removal of the IUD, thereby providing a useful reference for the diagnosis and treatment of similar cases in the future. Furthermore, the patient’s clinical management fully demonstrated the importance of a multidisciplinary approach involving gynaecology and urology in the management of such complex cases.

IUD migration is relatively rare. To avoid causing unnecessary harm to patients, clinicians must strictly adhere to the indications and contraindications for IUD insertion, be proficient in the surgical technique and postoperative precautions, and exercise caution during the procedure to minimise the risk of serious complications such as uterine perforation and IUD migration. Patients with an IUD *in situ* should be advised to attend regular follow-up appointments. If a patient presents with persistent urinary tract symptoms or recurrent urinary tract infections, IUD migration should be suspected, particularly when the IUD cannot be visualised within the uterine cavity. For patients in whom imaging studies suggest IUD migration, a personalised treatment plan should be implemented as early as possible according to the location of the migrated device. The prognosis for such patients is favourable.

## Patient consent

The patient expressed relief at obtaining a definitive diagnosis after 2 years of unexplained urinary symptoms. She emphasised the importance of fertility-preserving surgical strategies and reported satisfaction with the multidisciplinary treatment approach. She provided written consent for publication and wished to raise awareness regarding routine IUD surveillance. We thank the patient for permitting us to use her data to complete this article.

## Data Availability

The original contributions presented in the study are included in the article/supplementary material, further inquiries can be directed to the corresponding authors.
